# A Study of the Interface of Gold Nanoparticles Conjugated to Cowpea Fe-Superoxide Dismutase

**DOI:** 10.3390/antiox11112082

**Published:** 2022-10-22

**Authors:** Edurne Tellechea, Aaron C. Asensio, Paula Ciaurriz, Javier Buezo, Pedro López-Gómez, Marina Urra, Jose F. Moran

**Affiliations:** 1 NAITEC-Technological Center of Automotive and Mechatronics, C/Tajonar 20, 31006 Pamplona, Spain; 2 Institute for Multidisciplinary Research in Applied Biology (IMAB), Department of Sciences, Public University of Navarre (UPNA); Avda. de Pamplona 123, 31192 Mutilva, Spain

**Keywords:** biofunctionalization, gold nanoparticle, superoxide dismutase, SOD

## Abstract

The iron superoxide dismutase (FeSOD) is a first barrier to defend photosynthetic organisms from superoxide radicals. Although it is broadly present in plants and bacteria, FeSODs are absent in animals. They belong to the same phylogenic family as Mn-containing SODs, which are also highly efficient at detoxifying superoxide radicals. In addition, SODs can react with peroxynitrite, and FeSOD enzyme has already been used to evaluate the anti-nitrative capacity of plant antioxidants. Gold nanoparticles (AuNPs) have been shown to significantly improve the functionality and the efficiency of ligands, providing they are properly assembled. In this work, the characteristics of the recombinant cowpea (*Vigna unguiculata*) FeSOD (r*Vu*FeSOD) immobilized onto AuNPs were investigated as a function of (1) NP surface chemistry and (2) biofunctionalization methods, either physical adsorption or covalent bonding. The NP surface chemistry was studied by varying the concentration of the ligand molecule 11-mercaptoundecanoic acid (MUA) on the NP surface. The coverage and activity of the protein on AuNPs was determined and correlated to the surface chemistry and the two biofunctionalization methods. rVuFeSOD–AuNPs conjugate stability was monitored through absorption measurements, agarose gel electrophoresis and DLS, enzymatic activity by a colorimetric assay and by in-gel activity assay, and coverage was measured by colorimetric assay. When using physical adsorption, the NP is the most perturbing agent for the activity of the enzyme. In contrast, only the NP coverage was affected by MUA ligand concentration. However, during covalent attachment, both the NP and the concentration of MUA on the surface influenced the enzyme activity, while the coverage of the NP remained constant. The results evidence the importance of the biomolecule and AuNP interaction for the functionality of the hybrid. These strategies can be used to develop electrochemical biosensors for O_2_^•−^ and for peroxynitrite in biomedical applications.

## 1. Introduction

Superoxide dismutases (SODs; superoxide:superoxide oxidoreductase, EC 1.15.1.1) are an essential group of metalloenzymes that have been crucial for the evolution and maintenance of oxygenic life. They represent a first line of defense against free radical production from oxygen by specifically catalyzing the dismutation of superoxide radicals (O_2_^•^^−^). SODs catalyze two-step reactions in which two superoxide anions dismutate to hydrogen peroxide and water, while a metal cofactor cycles between its reduced and oxidized forms [1]. Superoxide dismutation by SODs is among the fastest enzyme reactions known [2], and is only limited by diffusion velocity. SODs are found in all three domains of life, and may well have been present in LUCA (the last universal common ancestor of life), long before the existence of an oxygenic atmosphere on earth, to break the O_2_^•−^ produced from the UV-radiation-derived splitting of water. Virtually all organisms that spend any time in the presence of oxygen possess copies of this enzyme. Three major families of superoxide dismutase are currently known, and these families depend on the metal cofactor: the Cu/Zn type, the Ni type and the Fe and Mn type [2]. 

In every living organism, the regulation of O_2_^•−^ production and elimination is very important. The excess O_2_^•^^−^ are involved in many physiological and pathological processes. O_2_^•^^−^ is strongly correlated with tissue injury and associated inflammation, and certain cancers and aged–related disease, such amyotrophic lateral sclerosis, Parkinson’s disease, Alzheimer’s disease, cataracts and various neurological disorders [2]. Therefore, the monitorization of superoxide anions in biological systems, both in vivo and in vitro, is of great importance to reveal and counteract O_2_^•^^−^ physiological functions. Superoxide radicals are highly reactive and short-lived, and therefore difficult to detect in complex biological matrices. In recent years, different detection techniques have been developed, i.e., electron paramagnetic resonance (EPR) [3], surface-enhanced Raman spectroscopy (SERS), fluorescence imaging, photoprotein-based bioluminescence, and electrochemical biosensors [4]. Considerable attention has been paid to the direct electrochemistry of SOD and related O_2_^•^^−^ detection [5] as a non-damaging, low-cost, and effective selective technique, but it requires the immobilization of SOD onto the electrode surface. Several strategies have been employed to improve electron transfer between SOD redox enzyme and electrodes, and it appears a promising way to use nanomaterials as the protein carriers. These hybrid electrodes can be built on a nanometric scale at low cost, and with strong analytical response. The electrode surfaces are chemically and physically diverse, and they include CeO_2_ nanoparticles [6], Pt–Pd multiwalled carbon nanotubes [6] (MWCNTs), ZnO nanoparticles [7], and gold nanoparticles (AuNPs) [8,9,10,11], AuNPs being one of the most studied nanomaterials. Another interesting application of SOD–AuNP complexes is the development of highly sensitive colorimetric detection of the temporal evolution of SOD1 aggregates implicated in the pathology of amyotrophic lateral sclerosis (ALS) [12]. Additionally, AuNPs have been described as a new class of surface immobilized pH-stimulus-responsive hybrid material based on tethered SOD1 [13], and as SOD carriers for possible therapeutic applications [14].

Many immobilization methods have been developed for SOD and AuNPs, such as direct binding, encapsulation, and crosslinking, although the most popular biofuctionalization method is noncovalent immobilization [5,6,7,10]. CuZnSOD (SOD1) is the most frequently used SOD [8,9,10,12,13]), while FeSOD, is the least studied group of the main SOD families [15,16]. Generally, these studies focus on the effect of whole conjugates (SOD–AuNPs) on the application (i.e., electrochemical measurements). While some enzymes retain their activity once they are localized upon the AuNPs [14,17], others are strongly affected [15]. In some cases, the NPs can improve enzyme activity and stability [18]. Moreover, it has been observed that the ligand used to passivate nanoparticles also plays an important role in enzyme activity [15]. Thus, the characterization of the VuFeSOD enzyme interaction with AuNPs, and the stability of the resulting particle complexes, is of critical importance to many functional applications.

Here we investigate the characteristics of a recombinant (r*Vu*FeSOD) protein from the legume cowpea (*Vigna unguiculata*) immobilized on 9.6 nm AuNPs. Cowpea is an important legume crop originating from tropical and subtropical areas. It is of increasing agronomic interest due to its ability to tolerate drought. Cowpea FeSOD has been shown to participate in the response to drought stress by eliminating superoxide radicals [19]. Within eukaryotes, the FeSOD family is typically found in chloroplasts, with few exceptions, the most important being the FeSOD of the legume plants. Thus, VuFeSOD belongs to a second family of FeSODs localized in the cytosol, and they are more important than the chloroplastic family in terms of biological activity within the cells [19,20]. VuFeSOD has been crystallized [21], its 3D structure has been solved, and the model refined to 1.97 Å resolution [22]. FeSOD is a homodimer enzyme with one binding site for superoxide anions per subunit, which is located at a cleft close to the dimer interface. The dimer interface also shows that some amino acids are considered to play an important role in the catalysis due to subunit cooperation [22]. rVuFeSOD plays a role in symbiotic nodules during biological fixation of atmospheric nitrogen, where it can protect the cytosolic symbiotic hemoglobin from oxidation with superoxide. The *Vu*FeSOD contents in the plant cells increase with the age of the plant, and VuFeSOD also participates in the plant response to abiotic stresses such us drought, salt, high irradiance, and others, which limits field agronomic production. This protein has a high affinity for the superoxide anion, and it is possible to overexpress and purify it in very high concentrations [23].

In addition, VuFeSOD has been used as a sensor of the harmful peroxynitrite anion, since peroxynitrite targets important metalloproteins, as superoxide dismutases, with high affinity [24,25,26]. In this respect, VuFeSOD has been used to test, in vitro, the anti-nitrative and protective capacity of different antioxidants from plants [26,27]. Peroxynitrite also targets hemoproteins and other metalloproteins with high affinity [27]. Hence, VuFeSOD bound to AuNPs can be useful as an affordable and specific sensor to detect either superoxide or peroxynitrite radicals, or to protect hemoglobins and other proteins from oxidation. There is a broad array of biomedical applications that can benefit from this technology, including different medical situations, such as inflammation, cardiovascular diseases, or hypoxia–reperfusion injury.

Here we show that the r*Vu*FeSOD enzyme can conjugate to AuNPs covalently and noncovalently, using mercaptoundecanoic acid as a ligand. The effect of ligand concentration on protein activity and coverage was determined to ascertain its correlation with surface chemistry. Our results show that both the surface chemistry and how the enzyme interacts with the NP, either covalently or by physical adsorption, affects the activity and coverage. These results improve the FeSOD utility and efficiency in its range of applications, potentially enhancing, for example, the capabilities of electrochemical nanosensors for SOD detection or even biomedics, with nanodeposition of Au particles via FeSOD in tissues where pathologic SOD production is typically detected, e.g., in anoxia–reperfusion injuries.

## 2. Materials and Methods

### 2.1. Chemicals and Biological Material

All chemicals were purchased from Sigma-Aldrich Co. (St. Louis, MO, USA), except when indicated. HisTrap HP 5 mL chromatography columns were supplied by GE Healthcare (Uppsala, Sweden). The buffer used with the protein contained 10 mM sodium phosphate (NaP) and was adjusted to pH 7.4. MilliQ Water (Millipore, Bedford, MA, USA) was used throughout this study.

### 2.2. Methods

#### 2.2.1. Overexpression, Purification and Activity of Recombinant VuFeSOD

The *Vu*FeSOD was recombinantly engineered into pET28a+ as described previously [19]. Overexpression and purification of the recombinant *Vu*FeSOD was established by the optimized method, as reported previously. Protein purification was assessed by gel electrophoresis (10% PAGE, *w*/*v*), and purified protein aliquots were pooled and stored at −20 °C until used. In order to quantify total protein, Bradford dye binding assay was used with bovine serum albumin (BSA) as a standard. Enzymatic activity of SOD was assayed on 12.5% (*w*/*v*) native acrylamide gel [28].

#### 2.2.2. Gold Nanoparticles Synthesis (AuNPs) 

Water-soluble, citrate-stabilized AuNPs (d = 9.6 ± 2.2 nm) were synthesized according to literature methods [29]. Briefly, 80 mL of 1% tetrachloroauric acid (HAuCl_4_) was reduced by adding a mixture of 4 mL of 1% sodium citrate (*w*/*v*), 0.1 mL of 1% tannic acid (*w*/*v*), 1 mL of 25 mM sodium carbonate, and 14.9 mL of H_2_O at 60 °C under vigorous stirring. At the beginning, the color of the solution was purple, but changed to red as the reaction continued. After 10 min at stable temperature, the colloidal solution was cooled down at room temperature. 

After the synthesis was completed, transmission electron microscopy (TEM) images were captured to evaluate size distribution of the particles. The average size of the particles was obtained by analysis of TEM images JEOL 2011 (JEOL, Inc., Peabody, MA, USA) with ImageJ software (https://imagej.net, accessed on 16 May 2022) [30].

The concentration of the AuNP solution was calculated by absorption spectrophotometry. The AuNPs (9.6 nm) showed an absorption peak at 517.5 nm. Gold nanoparticles were assumed to possess the bulk structure of gold, and a population of atoms per volume unit (~5.9 × 10^28^ m^−3^) was reported previously [31]. Accordingly, the 9.6 nm AuNPs in our study were composed of approximately 27,332 atoms with an extinction coefficient of about 9.1 × 10^7^ cm^−1^ × M^−1^ [32]. The concentrations of certain AuNP solutions were then calculated via the Lambert–Beer law, where absorbance at 800 nm is subtracted from the peak absorbance as a baseline value (Equation (1)).

(1) $$C=\frac{Abs_{517.5nm}-Abs_{800nm}}{\varepsilon_{abs}}$$

#### 2.2.3. Surface Modification of AuNPs

The surfaces of the NPs were modified with mercaptoundecanoic acid (MUA) using different NP:ligand stoichiometry: 1:100, 1:250, 1:500, 1:750, and 1:1000. MUA contains a thiol group in one side of the chain that facilitates a covalent bond to Au and a carboxyl group on the other side. Gel electrophoresis, size, and UV–Vis spectra were used to assay the surface chemistry. Agarose gel electrophoresis (0.5%) was performed in Tris-acetate (TA) buffer at E = 80 mA/cm for 60 min.

#### 2.2.4. Conjugation of SOD to AuNPs

Two different approaches were used to conjugate the protein to nanoparticles. The first approach used physical adsorption between the particle and protein to generate supramolecular assemblies; the second used covalent linkage of the protein to the particle by means of stable amide bond formation between the carboxylic group of MUA and protein. 

Protein adsorption: NP–r*Vu*FeSOD conjugation was achieved by incubating r*Vu*FeSOD with the NPs in solution at room temperature for 1 h. The incubation ratio was 1:500 NP:r*Vu*FeSOD, which was chosen based on the titration method. This ratio most likely represents a large excess of protein; thus, it helps to ensure conjugation. NP–r*Vu*FeSOD conjugates were purified by centrifugation at 4 °C. 

Covalent linkage: an initial activation of the carboxylic groups of MUA was accomplished by loading the NPs with freshly prepare activation solution EDC/sulfo-NHS (ratio 2:1). EDC (1-ethyl-3-(3-dimethylaminopropyl) carbodiimide hydrochloride) is a water-soluble zero-length carbodiimide crosslinker that activates carboxyl groups for spontaneous reaction with primary amines, enabling protein conjugation. The addition of sulfo-NHS (*N*-hydroxy sulfosuccinimide) to reactions increases the efficiency and enables the carboxylic group to be activated for longer period of time [32]. The activation solution contained different concentrations of EDC/sulfo-NHS for every NP:MUA stoichiometry, and it depended on the concentration of carboxylic groups on the nanoparticle surface. The ratio COOH:EDC was always 1:10. After 1 h of incubation, the nanoparticles were washed with coupling buffer using centrifugation and resuspended using a sonic probe. The proteins were introduced in the solution to produce the covalent attachment via ester linkage. After 2 h of incubation, NP–r*Vu*FeSOD conjugates were purified by centrifugation at 4 °C. The purified conjugates were finally dispersed in buffer and storage at 4 °C. Surface chemistry was assay by agarose gel electrophoresis (0.5%) in TA at E = 80 mA/cm for 60 min. Enzyme coverage was quantified by the Bradford protein assay using BSA as standard. The number of r*Vu*FeSOD per NP was obtained from the difference between the initial and final protein concentrations in solution, divided by the initial NP concentration.

#### 2.2.5. Superoxide Dismutase In-Gel Activity Assays for the Conjugates

In-gel SOD activity assays for AuNP–r*Vu*FeSOD conjugates were performed by electrophoresis on agarose gels (0.5%) in 0.5× Tris-acetate buffer pH 8 at 75 V for 90 min. After the electrophoresis, the gels were stained according to [28], with the following modifications. Agarose gels were kept in the buffer (50 mM sodium phosphate, pH 7.8) for 30 min. Secondly, the gel was transferred to 0.5 mM nitroblue tetrazolium-containing buffer, and it was further incubated for 20 min. Finally, they were incubated in the reaction buffer supplemented with 0.03 mM riboflavin and 0.2% tetramethylethylenediamine (TEMED) (*v*/*v*) for 20 min. All incubations were performed in darkness. Gels were then exposed to white light for 2–5 min to visualize SOD activity bands. 

#### 2.2.6. Spectrophotometric Activity Assay

The enzymatic activity of r*Vu*FeSOD was spectrophotometrically quantified, based on the ability of SODs to inhibit NBT reduction by the xanthine + xanthine oxidase (XOD) system [33]. Reduction in NBT was monitored at 25 °C by following the increase in absorbance at 560 nm for 5 min. The reaction cocktail contained reaction buffer (10 mM potassium phosphate, 0.1 mM Na_2_EDTA, pH 7.4), 0.1 mM xanthine, and 0.25 mM NBT. The reaction was started by the addition of 40 μL of 1:1600 diluted XOD. Sample volumes of NP–r*Vu*FeSOD conjugates were adjusted to reduce activity rate to around 50%. Subsequently, 10–20 µg of r*Vu*FeSOD was finally employed. A sample without XOD was used as a blank (negative control). Data from different syntheses and experimental replicates were collected to calculate the average (minimum n = 3).

#### 2.2.7. Dynamic Light Scattering

Size measurements were performed by dynamic light scattering (DLS) with a Zetasizer Nano series instrument (Malvern Instruments, Worcestershire, UK). Nanoparticle average hydrodynamic diameter (intensity) as well as the polydispersity index (PdI) were measured before and after conjugation. DLS measurements were performed using the following settings: laser wavelength of 633 nm (He–Ne) and scattering angle 90°; measurement temperature 25 °C, medium viscosity 0.8872 mPa·s, and medium refractive index 1.330. All the samples were sonicated before measurement and standard 1 mL disposable polystyrene cuvettes were used. 

## 3. Results and Discussion

### 3.1. Engineering the NP Surface Chemistry 

**Figure 1:**
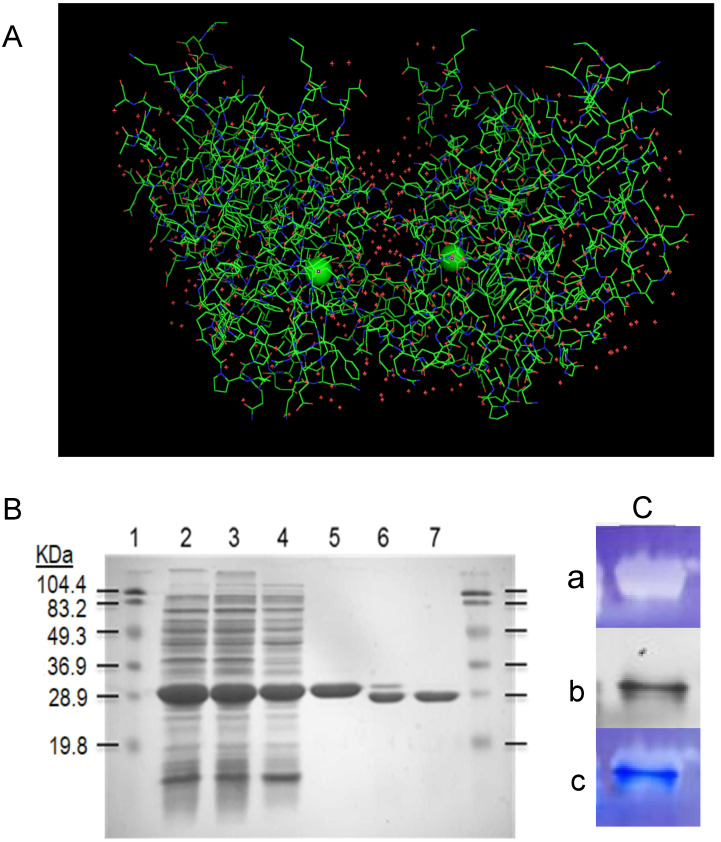
(**A**) *Vu*FeSOD dimer crystal structure: 3D model for the crystal structure dimer of the eukaryotic FeSOD from cowpea nodule cytosol, based on X-ray crystallography of FeSOD crystals (http://doi.org/10.2210/pdb1UNF/pdb accessed on 16 October 2022). Iron atoms are presented as green circles. (**B**) SDS-PAGE gel of the r*Vu*FeSOD purification steps: samples were electrophoresed in a 12.5% SDS-PAGE gel and stained with Coomassie Blue. Line 1: Molecular mass markers (KDa); Line 2: Crude extract, Cell disruption; Line 3: Crude extract, pellet; Line 4: Crude extract, supernatant; Line 5: Pure r*Vu*FeSOD; Line 6: Pure r*Vu*FeSOD partially digested with thrombin; and Line 7: Pure r*Vu*FeSOD digested with thrombin. (**C**) r*Vu*FeSOD gel activity: (a) Gel stained for SOD activity assay (~2 µg of protein), (b) replica gel stained with Coomassie blue brilliant, and (c) superposition of gel stained with Coomassie and gel stained for SOD activity.

r*Vu*FeSOD is a homodimeric enzyme with one noncovalently bound iron atom co-factor per monomer (Figure 1A). The r*Vu*FeSOD monomer has 245 amino acid residues, seven α-helices, and three β-sheet structures. Its overall size is ~45 × 31 × 36 Å, the isoelectric point is 5.31, and the molecular weight is 27,467 Da, including one atom of Fe per monomer. Additionally, the 3D model obtained by X-ray diffraction is available [21,22]. The r*Vu*FeSOD was produced and purified according to the protocol described in the Materials and Methods of [23]. The protein was produced in a soluble state, with little or no inclusion bodies. The purification steps of r*Vu*FeSOD are shown in Figure 1B. Activity of r*Vu*FeSOD was checked using gel stained for SOD activity assay. After electrophoresis on native polyacrylamide gels and staining, the negative banding corresponding to the SOD activity was visualized (Figure 1C).

**Figure 2:**
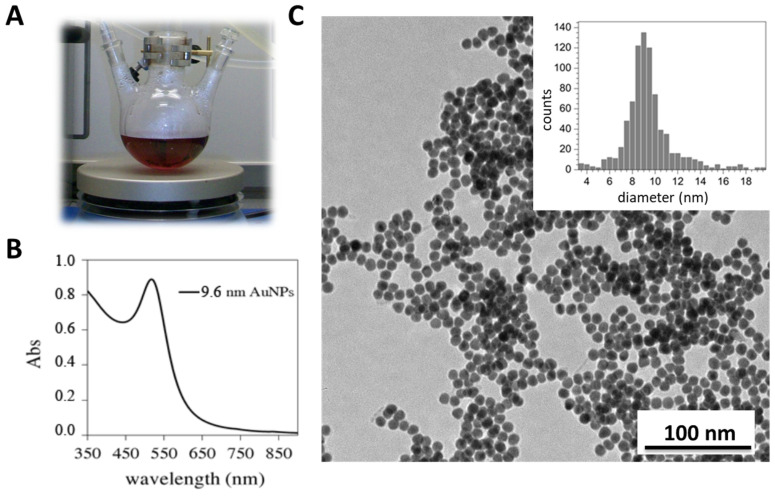
Synthesis of 9.6 nm gold nanoparticle: (**A**) reduction in tetrachloroauric acid in the presence of a reducing agent (citrate) to form colloid AuNPs (red solution); (**B**) UV–Vis absorption spectra of AuNPs with an absorption peak at 517.5 nm; and (**C**) TEM images of AuNPs and their respective size distribution histograms.

Citrate-stabilized AuNPs were synthesized (Figure 2A—red solution). AuNP present the localized surface plasmon resonance (LSPR), which refers to the collective oscillation of electrons within the material when they are excited by light [34], giving rise to a characteristic absorption peak (Figure 2C) that varies depending on the AuNPs size, form, and surrounding medium [35]. Transmission electron microscopy (TEM) images showed spherical shaped particles with an average diameter of 9.6 ± 2.2 nm, while dynamic light scattering (DLS) measurements revealed an average hydrodynamic diameter (intensity) of 11.3 ± 0.6 nm (Figure 2). As expected, the DLS size was slightly larger than in TEM, since the latter corresponds to a dry particles size. NP surfaces were modified with 11-mercaptoundecanoic acid (MUA, SH-(CH_2_)_11_-COOH). MUA is well known to bind to the gold atoms by the -SH group, and the carboxylic terminal group is relatively easy to bind covalently to the protein. Different amounts of surface coating MUA ligand were used so that the effect of NP surface chemistry on r*Vu*FeSOD behavior could be studied. We used NP−citrate as the starting material, and then modified it with different concentrations of MUA. Under these conditions, MUA is well known to bind to gold atoms by the -SH group. 

We probed the following NP:MUA stoichiometries: 1:100, 1:250, 1:500, 1:750, and 1:1000 to assess the density of the packing. The packing density of MUA onto spherical gold nanoparticles has been measured previously. Thus, it was determined that the packing density for AuNPs modified with a MUA was 4.97 ± 0.01 ligand molecules∙nm-2 [36], while other investigators previously reported a value of 5.7 ± 0.13 [37]. Since the packing density of MUA is nanoparticle-size-independent, the highest possible packing on the 9.6 nm AuNPs modified with MUA is in the range of 1450–1650 molecules per nanoparticle. Therefore, our particles are not totally covered by the SAM. 

**Figure 3:**
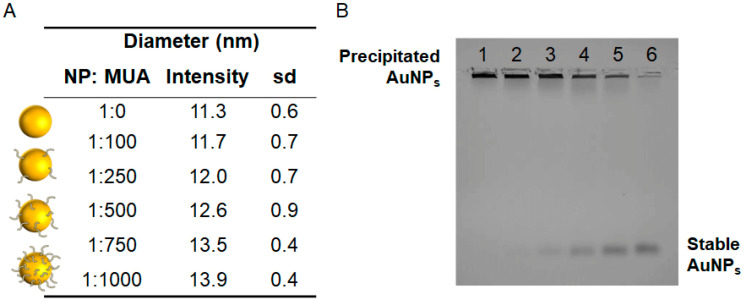
AuNPs surface modification with different concentrations of MUA. (**A**) AuNPs hydrodynamic size measured by Zetasizer in water. The diameter (intensity) increases linearly with MUA concentration on the surface. (**B**) Gel electrophoresis mobility shift of NP–citrate modified with MUA: Lanes: (1) NP–citrate, (2) 1:100 NP:MUA ratio, (3) 1:250 NP:MUA ratio, (4) 1:500 NP:MUA ratio, (5) 1:750 NP:MUA ratio, and (6) 1:1000 NP:MUA ratio.

NPs with different surface ligand coatings presented slight differences in hydrodynamic diameter. The hydrodynamic diameter increased from 11.30 to 13.94 nm (Figure 3A). Gel electrophoresis also showed mobility shifts that varied with surface chemistry (Figure 3B). NP−citrate was not stable enough in the buffer to run in the gel (lane 1), which has been observed previously [38]. MUA functionalization increased the NP mobility in gel electrophoresis (from lane 2 to line 6), which may be due to the negative charge from MUA. As we increased the concentration of MUA on the NPs surface, the NPs began to gradually become more stable in the gel [39], run more homogeneously, and shifted to lower mobility, most likely due to the increase in size. 

### 3.2. Gold Nanoparticle Protein Conjugation

**Figure 4:**
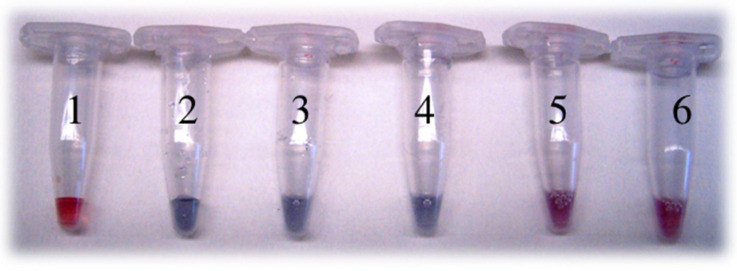
Determination of the optimal NP:r*Vu*FeSOD ratio: Lines (1) Citrate AuNPs, (2) 1:1 Citrate AuNP:r*Vu*FeSOD, (3) 1:10 Citrate AuNP:r*Vu*FeSOD, (4) 1:100 Citrate AuNP:r*Vu*FeSOD, (5) 1:500 Citrate AuNP:r*Vu*FeSOD, and (6) 1:1,000 Citrate AuNP:r*Vu*FeSOD. The lowest amount of protein that maintained a stable colloidal solution after addition of NaCl was selected, corresponding to 1:500 AuNP:r*Vu*FeSOD (tube #5).

**Figure 5:**
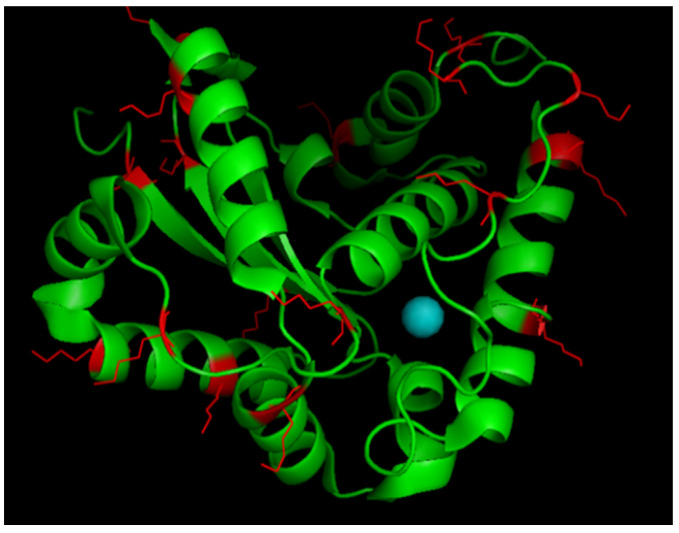
3D model for a monomer of *Vu*FeSOD: Lysine residues are presented in red.

NPs with different surface chemistries were then conjugated to r*Vu*FeSOD. Two different approaches were used to conjugate the protein to nanoparticles. The first approach used physical adsorption of the protein to the particle surface. For this purpose, conjugation was achieved by simple incubation for 1 h at a ratio of AuNPs to r*Vu*FeSOD of 1:500. Determination of the optimal NP:r*Vu*FeSOD ratio was achieved using the titration method [40] (Figure 4). Unbound r*Vu*FeSOD was separated by spin centrifugation. The second method used direct covalent linkage of the protein to the particle surface. The covalent attachment involves a series of consecutive steps. First, activation of the MUA’s carboxylic groups was accomplished by loading the nanoparticles with activation solution EDC/sulfo-NHS [41]. In order to avoid the aggregation of NPs, we determined the activation conditions for each NP:MUA sample, to ensure the protein–AuNP linkage. Several ratios of EDC were revealed with each NP:MUA ratio. Finally, a 1:10 MUA:EDC ratio was chosen. AuNPs were activated for 1 h, and then the protein (1:500 NP: protein ratio) was loaded in the solution to produce the covalent attachment via active ester methods. After 2 h of incubation, AuNP–r*Vu*FeSOD conjugates were purified by centrifugation at 4 °C. r*Vu*FeSOD contains 18 lysine (-NH_2_) susceptible to form the covalent bond. They were distributed across the surface of the protein (Figure 5). We assumed that the junction between the MUA and protein will be random, having higher probability those residues located at the surface.

**Figure 6:**
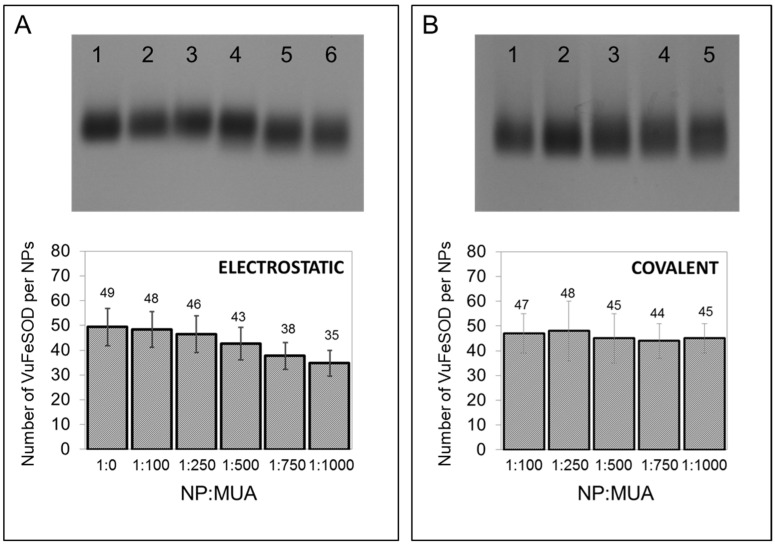
Effect of surface chemistry of AuNP-linked r*Vu*FeSOD on native gel electrophoresis and coverage: (**A**) Physical adsorption: Lanes: (1) NPs−r*Vu*FeSOD, (2) 1:100 NP:MUA–r*Vu*FeSOD, (3) 1:250 NP:MUA–r*Vu*FeSOD, (4) 1:500 NP:MUA–r*Vu*FeSOD, (5) 1:750 NP:MUA–r*Vu*FeSOD, and (6) 1:1,000 NP:MUA–r*Vu*FeSOD. (**B**) Covalent: Lanes: (1) 1:100 NP:MUA–r*Vu*FeSOD, (2) 1:250 NP:MUA–r*Vu*FeSOD, (3) 1:500 NP:MUA–r*Vu*FeSOD, (4) 1:750 NP:MUA–r*Vu*FeSOD, and (5) 1:1,000 NP:MUA–r*Vu*FeSOD.

Gel electrophoresis showed mobility shifts that varied with surface chemistry. The mobility shift is more pronounced for physical adsorption, rather than for covalent attachment. In physical adsorption, all the conjugates ran in the positive direction, indicating successful NPs stabilization and a net negative charge (Figure 6A). In addition, the narrow band indicated a uniform charge distribution, and thus uniform protein functionalization. The mobility of the conjugates increased with the concentration of MUA on the NP surface. The different mobilities can be explained by the protein coverage and by MUA concentration on the NPs surface. As we increase the negative charge of the nanoparticles with MUA, fewer proteins bind to the NPs, probably because r*Vu*FeSOD has a net negative charge at neutral pH, and the interaction between the negative particles and the protein becomes weaker. 

In covalent bonding, all the conjugates also ran in the positive direction, indicating a net negative charge (Figure 6B). They presented a similar narrow band. The mobility of conjugates was very similar for all samples, although it seems that the mobility slightly increases on the gel with MUA. Although covalent attachment of proteins to NPs provides conjugates that are stable toward dissociation, in our case, covalently binding nanoparticles presented a light trail of nanoparticles in the gel, which did not appear upon physical adsorption, indicating a greater heterogeneity of the samples.

For physical adsorption, the coverage was determined as 49 r*Vu*FeSOD per NPs−citrate. NPs−citrate modified with MUA progressively resulted in lower coverage 48 (1:100 NP:MUA), 46 (1:250 NP:MUA), 43 (1:500 NP:MUA), 38 (1:750 NP:MUA), and 35 (1:1000 NP:MUA), indicating that MUA inhibited conjugation (Figure 6). AuNPs modified with MUA are negatively charged particles; therefore, as we increase the MUA concentration on the nanoparticle, the nanoparticles surface become more negatively charged. All the bioconjugation assays were performed at the pH 7.4, at which r*Vu*FeSOD is also negatively charged (pI. 5.31). The repulsion tendency between negatively charged nanoparticles and negatively charged proteins starts to be more important as the negative charge on the NPs surface increases. These results correlate with those obtained in the gel electrophoresis.

For the covalent attachment, there were not significant differences on the sample to sample coverage: 47 (1:100 NP:MUA), 48 (1:250 NP:MUA), 45 (1:500 NP:MUA), 44 (1:750 NP:MUA), and 45 (1:1000 NP:MUA). The number of active carboxyl groups on the surface that we observed did not have any significant effect on coverage. These results also correlate with those obtained in the gel electrophoresis analysis.

We also checked the size of the conjugates with DLS (Table 1). We measured the size of the protein, and of the nanoparticles before and after conjugation. For physical adsorption, the size of the conjugates decreased as there was more MUA (carboxyl group) on the NP surface, which agrees with the gel electrophoresis and coverage results. For covalent bonding, the sizes slightly decreased with the number of carboxyl groups on the NPs surface. All the resulting conjugates presented a monolayer of protein lower than protein dimer hydrodynamic size (7.8 nm) indicating that our conjugates form a monolayer and not a multilayer. This result also agrees with the gel electrophoresis and coverage results. 

Conjugated stability was monitored via absorption measurements of the particles. Binding of the SOD on the AuNPs surface causes a small shift in the LSPR peak of the AuNPs. Absorption spectra for all conjugates showed that the LSPR peak underwent red shifts between 7 and 4 nm, accompanied by an increase in the intensity, indicating a proper bioconjugation. These results correlate with the DLS measurements.

**Figure 7:**
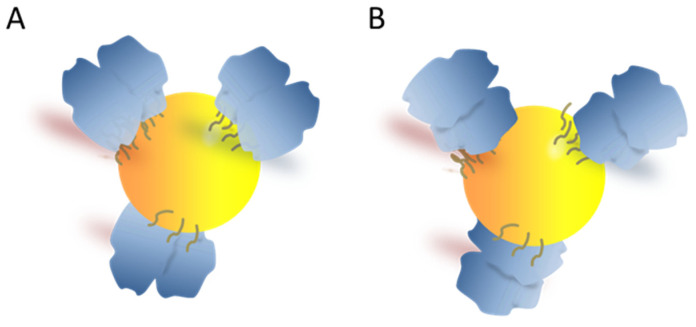
Different conformations of conjugates. (**A**) Both VuFeSOD monomers bind to the nanoparticles (**B**) one of the monomers binds to the NPs and the other does not.

Considering the previous results, the coverage values obtained were slightly higher than expected in the monolayer. We have estimated between 20–21 monomer proteins (10 full protein dimers, Figure 7A) per AuNPs as the maximum theoretical surface coverage for a 9.6 nm AuNP using crystallographic overall size of *Vu*FeSOD [21]. Depending on the protein arrangement, the theoretical number of proteins could be duplicated to 40 proteins (20 dimers) per AuNP, providing that one of the monomers binds to the NPs and the other does not (Figure 7B); however, it remains below the values obtained. The third possibility would be something intermediate between A and B. It is important to note that we calculated the concentration of r*Vu*FeSOD as a function of BSA calibration curve, therefore, these numbers probably are not the real coverage, but an estimate. Thus, NP surface chemistry was found to influence the coverage only when the protein was conjugated by physical adsorption.

**Figure 8:**
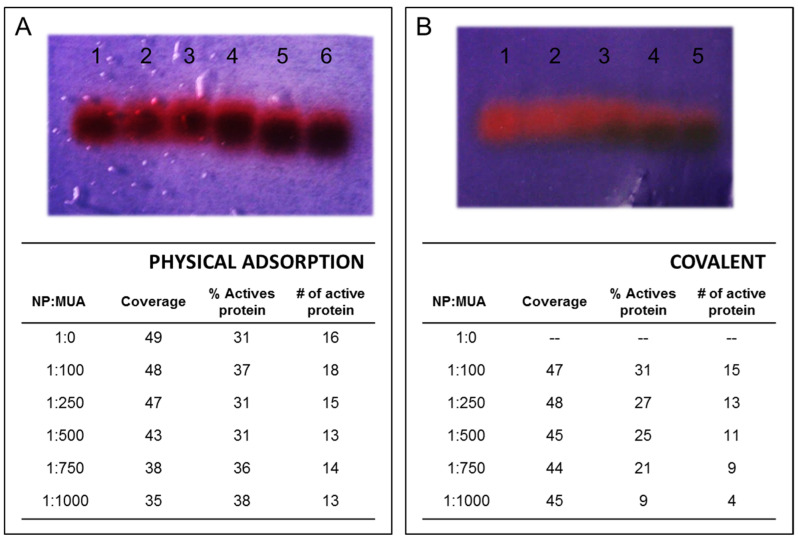
Effect of surface chemistry of AuNP-linked r*Vu*FeSOD on the superoxide dismutase activity assay outcome. (**A**) Physical adsorption: AuNP–*Vu*FeSOD activity gel for different NP:MUA stoichiometry and in the table, data of coverage, percentage of remaining superoxide dismutase activity, and number (#) of active proteins on the NPs. (**B**) Covalent attachment: NP–*Vu*FeSOD activity gel for different NP:MUA stoichiometry and in the table, data of coverage, percentage of remaining superoxide dismutase activity, and number of active proteins on the NPs.

To understand how MUA ligands impacted SOD function, the enzymatic activity of r*Vu*FeSOD on the different NPs was measured. First, SOD activity was measured in gels. Agarose gel was used to separate the NP–protein conjugates (Figure 8). During the physical adsorption process, a decrease in the activity in gel was observed as we increased the MUA concentration on the surface. Apparently, this may be related to the decrease in the coverage observed, and it would indicate that the −COOH groups did not necessarily affect the activity of the protein (Figure 8A). However, MUA caused a clear reduction in r*Vu*FeSOD activity onto AuNPs with covalent attachment (Figure 8B).

Secondly, an activity assay method was performed on the AuNP–*Vu*FeSOD solutions to assess the loss of activity after binding. For physical adsorption, the remaining active r*Vu*FeSOD on the NPs was in the range of 30–40%, indicating that r*Vu*FeSOD activity was compromised when bound to NPs. The activity measurements show that modifying citrate-stabilized NPs with MUA did not significantly influence r*Vu*FeSOD enzymatic activity, which indicates how strong a perturbing agent NPs are. Therefore, we can consider that the MUA molecule has not an effect on enzymatic activity of the protein, just on the coverage, and consequently, the coverage has no significant effect on the activity. 

Given that the retained activity of the r*Vu*FeSOD is lower than expected, we hypothesize that the protein behavior, which is based on a cooperative mechanism between the two monomers of the protein, is affected by the interaction of critical amino acids involved in the cooperative mechanism and the NPs [22]. In addition, at a physiological pH, SOD has a net negative charge. However, the deep and narrow active channel above the catalytic active site exhibits a highly positive charge that electrostatically attracts and guides the negatively charged O_2_^•−^ anions to the catalytic Fe metal at the bottom of the active channel [42]. This positive channel may be electrostatically attracted by the negative AuNPs surface, interfering or even blocking the access of O_2_^•−^ to the catalytic metal, and possibly exposing internal amino acids to enable binding to the NP. 

**Figure 9:**
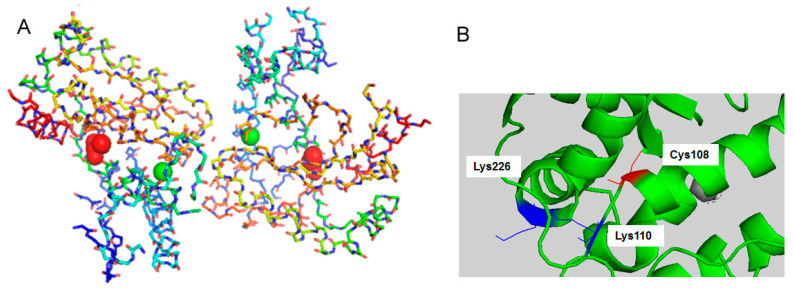
(**A**) VuFeSOD dimer crystal structure: 3D model for the monomer of the eukaryotic FeSOD from cowpea nodule cytosol, based on X-ray crystallography of FeSOD crystals. Iron atom presented as green circles and cysteine in red. The distance between the two Fe atoms is 18.01 Å and between the Fe atom and Cys from the same monomer 12.93 Å. (**B**) In red are the cysteine molecule (Cys108) of one monomer, and in blue are the two lysines that are near the Cys108: Lys110 and Lys226.

The r*Vu*FeSOD has only one Cys108 in each monomer. It is not on the surface of the protein, but relatively close to it (Figure 9); therefore, it may potentially bind to the nanoparticles, destabilizing the protein structure, similar to what it has been observed for yeast cytochrome *c* from *Saccharomyces cerevisiae* [43]. It is noteworthy that the cysteine molecule is neither at the interface of two proteins or near the active center (the FeCys distance is 12.93 Å), but quite near to His102, which is one of the four catalytic residues. Moreover, the cysteine molecule connects a loop with an alpha helix and is near two lysines (−NH_2_ containing), Lys110 and Lys226, which may interact with the carboxyl groups of the surface. Thus, it may be that the thiol from one monomer of r*Vu*FeSOD links to the NPs, forcing them to destabilize the folding of this monomer and disturb the dimer. In this situation, the protein involved may well lose an important part of its activity. It has been observed in *Escherichia coli* MnSOD (*Ec*MnSOD) that the disruption of the interaction between Tyr202 from one monomer with the neighboring monomer, His47, reduces the SOD activity to 30% to 40% [44]. In our case, the activity reduction is between 60–70%, indicating that a major form of disruption is taking place. It has been also indicated for FeSODs that have a cooperativity mechanism between the two monomers during catalysis. Indeed, in nature, FeSODs, and also MnSOD, are never monomers, they are always found as dimers or tetramers, but in the latter case they behave as a pair of dimers [22]. All these interactions may well explain the loss of 60–70% of the protein activity. In addition, the monolayer formed by the protein at its maximum coverage (DLS, 7.0 nm) is very close to the 7.8 nm of the protein diameter, supporting this hypothesis. 

For covalent bonding, the proportion of active r*Vu*FeSOD still present on the AuNPs was lower than 31%, indicating that r*Vu*FeSOD activity was especially compromised when covalently bound to NPs. This result points to the activation of carboxylic groups in MUA, as its interaction with Lys–NH_2_ residues is essentially implicated in the binding and loss of activity. Thus, the change in the MUA and NP surface chemistry induced a higher loss in protein activity. At this point, active r*Vu*FeSOD decreased when MUA concentration increased at the surface down to 9% at 1:1,000 NP:MUA, although the coverage was very similar in all conjugates. This suggests that increasing the probability of covalent linkage with highest MUA concentration reduces the stability of the r*Vu*FeSOD protein dimer. Similarly, it has been found that the covalent linkage of the CuZnSOD to a flat surface reduced SOD activity nearly 50% relative to the adsorbed state [16]. The coverage was very similar in all samples, but the monolayer size measured by DLS decreased slightly with MUA, suggesting that the surface caused the protein to change conformation without the possibility of a possible rearrangement of the protein. This conformational change has a direct impact on the activity.

## 4. Conclusions

In summary, a recombinant iron-superoxide dismutase from *Vigna unguiculata* (r*Vu*FeSOD) was conjugated to 9.6 nm AuNPs using either physical adsorption or covalent bonding. r*Vu*FeSOD was produced and purified to homogeneity in high yields using a self-induction overexpression system. In addition, 9.6 nm AuNPs with a high degree of control were synthesized, and their surfaces were modified with different concentrations of 11-mercaptoundecanoic acid ligand very easily.

Interactions with NPs can significantly alter the coverage and function of r*Vu*FeSOD. The NPs are generally a strongly perturbing agent for r*Vu*FeSOD, and the interactions between r*Vu*FeSOD and the NPs and NP ligands resulted in the significant inactivation of the protein activity. 

Different bioconjugation strategies produced different effects on the interaction between r*Vu*FeSOD and NPs. For physical adsorption, the impact of MUA concentration on the surface was more important in terms of coverage than activity. The MUA ligand acted more as a steric barrier that was also enhanced by the electrostatic repulsion, producing a reduction in coverage. However, the amount of MUA on the surface did not affect the activity, indicating that the strongly perturbing agent is the NPs itself. Evaluation of functional properties of the conjugate indicated that upon conjugation, proteins retained 31–39% of activity. 

For covalent attachment, however, the opposite effect was observed. The impact of MUA concentration was more important in terms of activity than coverage. As the concentration of MUA increased on the surface, the inactivation of the protein was higher; protein can lose 90% of its activity. This suggests that as well as the NP, the covalent linkage also destabilized the protein activity, probably due to an excess of attachment points to the surface. Overall, we have reported some remarkable results. The binding method, either physical adsorption or covalent linkage can help to improve or worsen the activity. For our system, physical adsorption has resulted in a better strategy. These results suggest that more optimization is needed for a better understanding of the interaction between r*Vu*FeSOD and AuNPs. Nevertheless, these strategies can be useful for applications, which require tethering the r*Vu*FeSOD and other enzymes to a surface. 

## Figures and Tables

**Table 1:** Protein layer thickness and LSPR shift (nm) of AuNP–VuFeSOD conjugates (physical adsorption and covalent linkage).

NP:MUA		1:0	1:100	1:250	1:500	1:750	1:1000	r*Vu*FeSOD (Dimer Size)
Protein layer thickness *	Physical adsorption	6.1	5.4	4.7	4.3	3.5	3.1	7.8
	Covalent	-	5.6	6.0	4.8	4.7	4.9	
∆ λ_LSPR_ (nm)	Physical adsorption	7.0	6.8	6.5	5.6	5.0	4.3	-
	Covalent	-	7.2	7.1	7.2	7.0	6.0	

* Protein layer thickness (DLS, intensity, nm).

## Data Availability

Data is contained within the article.
